# Safety and tolerability of agalsidase beta infusions shorter than 90 min in patients with Fabry disease: post-hoc analysis of a Japanese post-marketing study

**DOI:** 10.1186/s13023-023-02803-5

**Published:** 2023-07-24

**Authors:** Chae Sung Lee, Mina Tsurumi, Yoshikatsu Eto

**Affiliations:** 1 Sanofi, 450 Water Street, Cambridge, MA 02141 USA; 2 Sanofi K. K., Tokyo, Japan; 3 Advanced Clinical Research Center, Southern Tohoku Research Center for Neuroscience, Kanagawa, Japan

**Keywords:** Fabry disease, Enzyme replacement therapy, Agalsidase beta, Infusion time, Infusion-associated reaction

## Abstract

**Background:**

Agalsidase beta, an enzyme replacement therapy for Fabry disease, is dosed biweekly at 1 mg/kg body weight, with increasing infusion rates based on tolerability. The US label specifies ≥ 90-min infusions for all patients; the US and EU labels require ≤ 15 mg/hr infusions in patients < 30 kg. The Japanese label allows infusions up to 30 mg/hr, allowing < 90-min dosing for some patients weighing < 45 kg. Japanese post-marketing data were analyzed for rate of infusion-associated reactions (IARs), adverse events (AEs), and serious AEs (SAEs) based on infusion rate and patient attributes (weight, antibody status).

**Results:**

Data were available for 436 reduced-duration infusions (< 90 min) and 2242 standard infusions (≥ 90 min). SAEs were rare (0.6%), and the frequency of all safety events decreased over the treatment course. Little impact of infusion duration on safety outcomes was observed: IARs and AEs were numerically more common when infusion duration was ≥ 90 min compared to < 90 min (IARs: 2.0% vs 0.9%; AEs: 2.9% vs 1.4%), while the rate of SAEs was similar (0.4% vs 0.5%). IAR, AE, and SAE frequencies decreased significantly with increasing infusion rates, and this trend was consistent in patients < 30 kg. Safety events tended to be less frequent in patients < 30 kg vs those ≥ 30 kg (IARs: 1.8% vs 2.1%; AEs: 2.3% vs 3.6%; SAEs: 0.0% vs 0.6%), although the differences were not statistically significant. IARs occurred in < 1% of all infusions in the < 30 kg group, 84% of which were < 90 min. More anti-agalsidase beta antibody-positive patients experienced IARs (41.9% vs 30.7%; *P* = 0.0445) and AEs (61.1% vs 49.3%; *P* = 0.0497) vs antibody-negative patients; however, there was no significant difference in the frequency of SAEs. In patients with available data, no changes in antibody status were observed after infusion durations were reduced to < 90 min.

**Conclusions:**

The results of this post-hoc analysis demonstrated no significant impact of infusion duration on safety outcomes, and no significant difference in outcomes between patients of different weights. These findings suggest that infusion times in patients who are tolerating treatment can, with careful monitoring, be gradually decreased.

## Background

Fabry disease is a rare, progressive, multisystemic, X-linked lysosomal storage disorder caused by a mutation of the gene encoding the enzyme α-galactosidase A (α-GalA), which results in partial or complete α-GalA deficiency [1, 2]. The deficiency in α-GalA activity prevents the breakdown of globotriaosylceramide (GL-3) and other substrates, causing them to accumulate and ultimately resulting in tissue damage and multiple organ impairment [1, 3]. Symptoms often begin in childhood and frequently include neuropathic pain, fever, hypohidrosis, gastrointestinal symptoms, cornea verticillata, and angiokeratoma [1, 4]. As the disease progresses, organ damage may become life-threatening, ultimately leading to a decreased life expectancy and greatly impaired QoL [1].

Current treatment guidelines for patients with Fabry disease recommend a multidisciplinary approach that includes the use of enzyme replacement therapy (ERT) in conjunction with symptomatic treatments and medications to prevent the progression of tissue injury [5]. Two forms of ERT are currently available for the treatment of Fabry disease: agalsidase alfa (Replagal®) and agalsidase beta (Fabrazyme®; a biosimilar of which [agalsidase beta BS] is also approved in Japan) [5, 6]. While ERT remains the standard of care in Fabry disease, chaperone therapy in the form of migalastat (Galafold®) was granted approval in 2016 and has demonstrated efficacy in patients with amenable *GLA* mutations (approximately 35% to 50% of in vitro mutations, although this proportion has come under question).[5, 7].

The lives of both patient and caregiver are greatly impacted by Fabry disease. One study found that 50% of surveyed patients with Fabry disease reported an impact of the disease on their work, resulting in 20% leaving their job [8]. A systematic review and meta-analysis that included 26 studies with detailed Fabry disease data found patient quality of life (QoL) to be impaired across all domains of the SF-36 and the EQ-5D, demonstrating a major physical and mental burden [9]. Given that Fabry disease frequently presents in childhood and early initiation of treatment is recommended by international guidelines, there is also an associated caregiver burden as has been demonstrated by frequent missed work days and impacts to caregiver mental health [5, 10, 11].

While ERT is a mainstay of Fabry disease treatment and confers significant benefits across a variety of disease measures, the need for lifelong, semimonthly infusions carries its own challenges for both patients and caregivers, including disruptions to daily living, impairments to school and work activities, and consequences to family and social life [5, 8, 11, 12]. When economic impacts are considered, the burden associated with ERT extends beyond the individual or family and into the greater healthcare system. The annual treatment cost per Fabry disease patient has been estimated at approximately €110,796 to €200,000 in the EU, while a Japanese study found a total lifetime per patient cost of almost 9 million JPY (approximately €6 million) [13–15].

Given the many challenges experienced by patients with Fabry disease and their caregivers as well as the high healthcare costs associated with the disease, any reduction in disease or treatment-related burden could be expected to have a positive impact. In several other disease areas, shortening infusion durations is a strategy that has been associated with significant QoL improvements, including positive effects on work and social life, as well as reductions in healthcare costs and resource use [16–18]. Similarly, optimizing treatment administration strategies in Fabry disease has proven to be a successful technique for improving patient and caregiver QoL, as demonstrated by the positive outcomes associated with introduction of home-based therapy on the Fabry disease patient population [8, 12]. Despite the improvements conferred by a home-based treatment approach, the time requirements for agalsidase beta infusion remain high; 15 mg/hr is recommended as the initial infusion rate for all patients and the maximum infusion rate for patients < 30 kg in the US and the EU, and a minimum infusion duration of 90 min is specified by the US label, regardless of patient weight [19, 20]. Previous versions of the Japanese product label included similar restrictions, however a recent label update now suggests that the infusion rate can be gradually increased beyond these thresholds once patient tolerance has been established.[21] Reduced agalsidase beta infusion times as short as 90 min have been found to be well-tolerated; however, durations < 90 min have only been studied in a case series published in 2021, which found good tolerability with infusion times reduced to 45 min in 6 adult patients (5 females) with Fabry disease [22, 23]. Overall, the safety and tolerability of < 90 min agalsidase beta infusions is not yet well established, and there is limited data assessing the propensity for infusion-associated reactions (IARs), adverse events (AEs), and serious AEs (SAEs) with differing infusion rates in the real world setting. Therefore, the objective of this post-hoc analysis was to examine rates of IARs, AEs, and SAEs in patients receiving infusions of varying durations and rates. This analysis also considered patient weight and antibody status and analyzed safety outcomes according to these variables.

## Methods

Post-hoc analyses of the long-term use safety data from the Special Drug Use Investigation of agalsidase beta study (Registry number: AGAL03004) and all-case surveillance data of patients enrolled in the Drug Use Investigation of agalsidase beta (Registry number: AGAL02904) were conducted to evaluate the safety and tolerability of infusion rate escalation among patients receiving agalsidase beta infusions in a hospital setting; details of these post-authorization safety studies have been described previously [24].

Selection criteria for patients enrolled in the Special Drug Use Investigation of agalsidase beta or the Drug Use Investigation of agalsidase beta have been previously published [24]. Briefly, Japanese patients with Fabry disease diagnosed by a physician according to deficient α-GalA activity or the identification of *GLA* gene mutation were eligible for inclusion. Details regarding agalsidase beta treatment, including the duration of treatment, total number of doses, mean dosing interval, time of administration, and average dose, were collected every 6 months until the end of the study.

The study was conducted in accordance with the Japanese regulatory requirements of the Good Post-Marketing Study Practice, ethical principles of the Declaration of Helsinki and the Institutional Review Board (IRB) regulations. The need for informed consent was waived as these safety studies were mandated by the Japanese regulatory authorities in accordance with the Law for Ensuring the Quality, Efficacy, and Safety of Drugs and Medical Devices (Pharmaceutical and Medical Device Act). The clinical study protocol and other study-related documents were reviewed and approved by the local or central IRB of study sites.

### Safety outcomes

Safety assessment outcomes measured were the incidence of AEs, including SAEs, and IARs. AEs were defined as any untoward medical occurrence in a patient administered study treatment, including those that did not necessarily have a causal relationship with study treatment. SAEs were defined as those AEs that were life-threatening or resulted in death, permanent or significant disability, inpatient or prolonged hospitalization, or congenital abnormality. IARs were defined as any AE occurring during any infusion or reported on the day after the infusion. The decision to discontinue treatment, as well as subsequent management of AEs (including switching to other treatments) was made by the individual treating investigator. IARs were evaluated and categorized by system organ class (SOC) and preferred term (PT) using MedDRA/J version 16.1.

### Statistical analysis

The safety analysis sets comprised all patients who were eligible for these two studies. Baseline demographics were summarized using descriptive statistics. The mean (standard deviation [SD] and 95% CI), and median (range) were calculated for continuous variables, and the frequency and proportion were calculated for categorical variables. The frequency of AEs was summarized descriptively overall, and for each individual event (by SOC and PT). The Fisher's exact test and the Chi-square test were used to determine significant or non-significant associations between categorical variables.

## Results

### Patient disposition and baseline characteristics

**Fig. 1:**
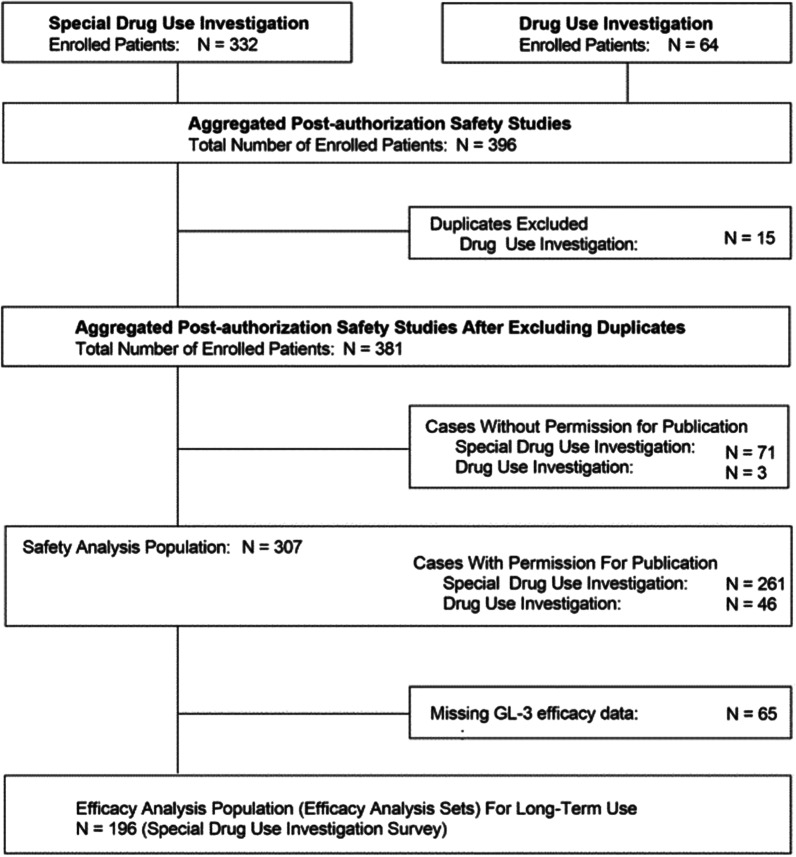
Study design and patient disposition. GL-3 = globotriaosylceramide

A total of 381 patients were enrolled in the safety study, with 307 comprising the safety analysis population after excluding those without permission for publication (Fig. 1). Patient demographics and baseline characteristics of each group are presented in Table 1. Demographics arranged by age and phenotype have been previously reported [24].

**Table 1:** Patient demographics and baseline characteristics

	Overall (n = 307)	< 30 kg (n = 6)	< 45 kg (n = 31)
Male, n (%)	201 (65.5)	5 (83.3)	19 (61.3)
Mean weight, kg (95% CI), [N]	54.7 (53.3–56.0), [254]	21.8 (16.7–26.8), [6]	36.8 (33.7–40.0), [31]
Mean age at initiation of ERT, years (95% CI), [N]	39.1 (37.3–40.9), [303]	7.3 (4.2–10.5), [6]	31.6 (23.2–40.1), [31]
Previous ERT use, n (%)			
No	234 (76.2)	5 (83.3)	24 (77.4)
Yes	71 (23.1)	1 (16.7)	7 (22.6)
NR	2 (0.7)	0 (0.0)	0 (0.0)
Previous concomitant medication use, n (%)			
No	27 (8.8)	0 (0.0)	0 (0.0)
Yes	280 (91.2)	6 (100%)	31 (100%)
Clinical symptoms at baseline, n (%)			
Pain in extremities	174 (56.7)	4 (66.7)	19 (61.3)
Angiokeratoma	95 (30.9)	0 (0.0)	6 (19.4)
Dyshidrosis	137 (44.6)	4 (66.7)	17 (54.8)
Dysthermesthesia	68 (22.1)	1 (16.7)	4 (12.9)
Corneal opacity	90 (29.3)	2 (33.3)	13 (41.9)
Abdominal pain	45 (14.7)	1 (16.7)	3 (9.7)
Diarrhea	54 (17.6)	1 (16.7)	5 (16.1)
Mean blood GL-3 level, μg/mL (95% CI), [n]	8.5 (7.7 − 9.4), [152]	10.7 (− 45.2 − 66.6), [2]	9.2 (6.5 − 11.9), [16]
Anti-agalsidase beta antibody status, n (%)			
Positive at any point during the study	167 (54.4)	3 (50.0)	17 (54.8)
Negative throughout the entire study period	140 (45.6)	3 (50.0)	14 (45.2)

ERT = enzyme replacement therapy; GL-3 = globotriaosylceramide

### Safety outcomes

#### Incidence of IARs, AEs, and SAEs

**Fig. 2:**
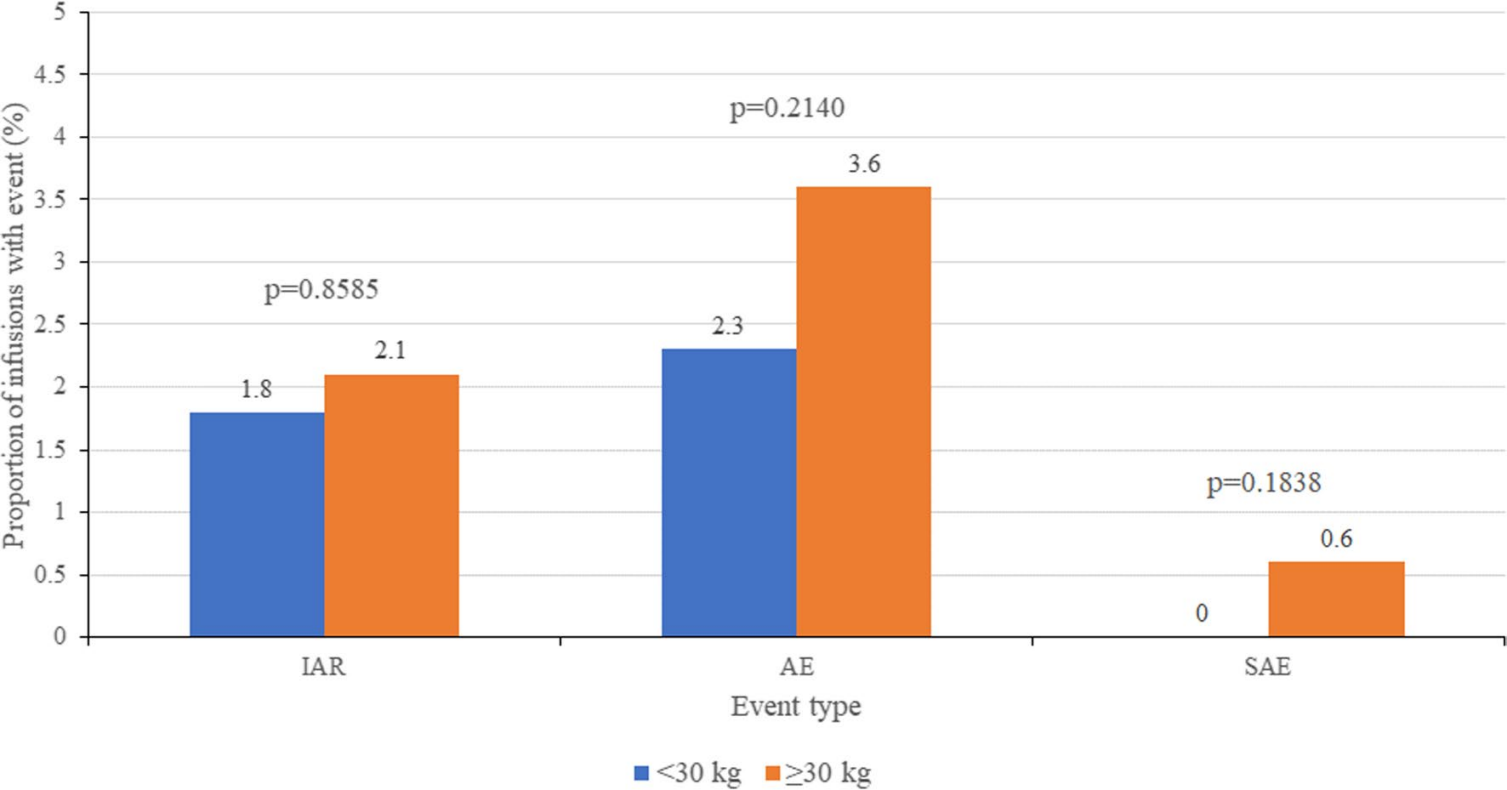
Frequency of IARs, AEs, and SAEs in patients ≥ 30 kg vs < 30 kg. AE = adverse event; IAR = infusion-associated reaction; SAE = serious adverse event

Overall, SAEs were rare (0.6%), and the frequency of all safety events decreased over the treatment course. In patients < 30 kg vs ≥ 30 kg, IARs (1.8% vs 2.1%, *P* = 0.8585), AEs (2.3% vs 3.6%, *P* = 0.2140), and SAEs (0.0% vs 0.6%, *P* = 0.1838) were numerically less frequent in patients < 30 kg (Fig. 2) despite a shorter mean infusion duration in the < 30 kg subgroup (88.7 ± 60.0 min, vs 178.2 ± 180.0 min in the ≥ 30 kg group), although the differences were not statistically significant.

#### Impact of infusion duration on safety outcomes

In the overall population, the proportion of patients who had an IAR was similar between those who had received at least one reduced-duration (< 90-min) infusion and those who only received standard ≥ 90-min infusions (42.0% vs 35.8%; P = 0.4257).

**Fig. 3:**
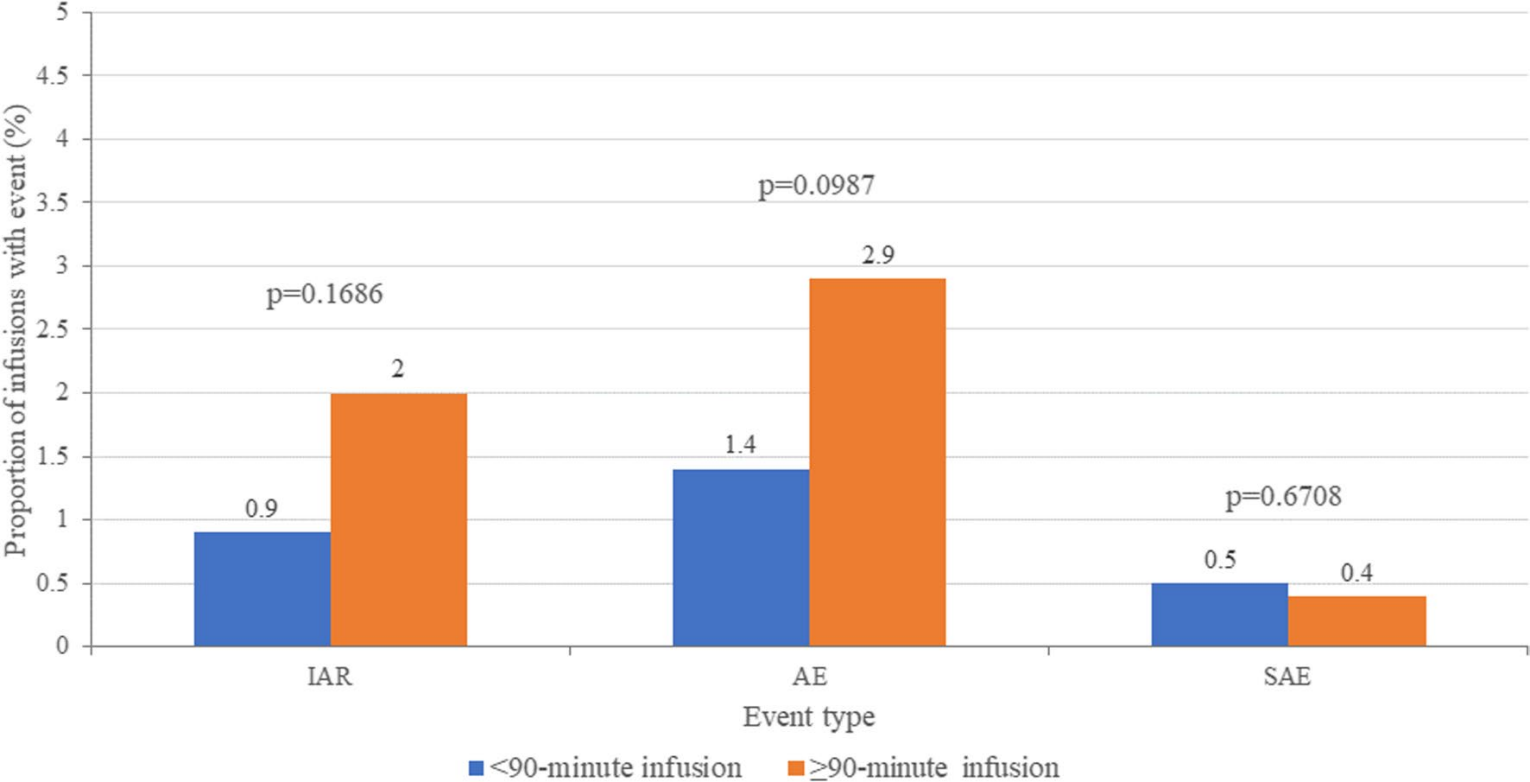
Frequency of safety events in the overall population according to infusion duration. AE = adverse event; IAR = infusion-associated reaction; SAE = serious adverse event

Data were available for 436 reduced-duration infusions (< 90 min) and 2242 standard infusions (≥ 90 min). Figure 3 compares the frequencies of safety outcomes (IARs, AEs, and SAEs) by infusion duration in the overall population. IARs and AEs were numerically more common when infusion duration was ≥ 90 min compared to < 90 min (IARs: 2.0% vs 0.9%; AEs: 2.9% vs 1.4%), while the rate of SAEs was similar (0.4% vs 0.5%). None of these differences were significant, demonstrating little impact of infusion duration on safety outcomes.

#### Impact of infusion rate on safety outcomes

**Fig. 4:**
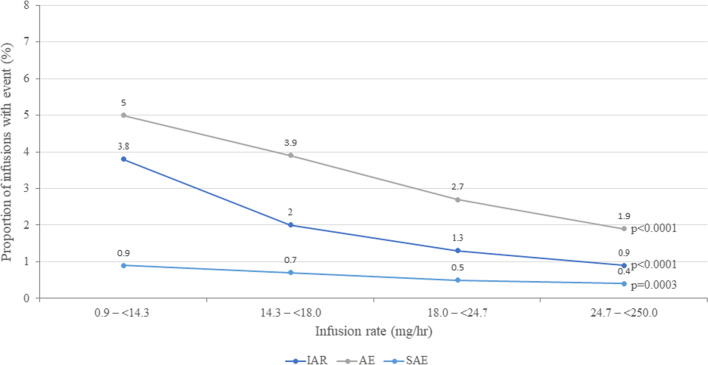
IAR, AE, SAE rates in the overall population with increasing infusion rates. AE = adverse event; IAR = infusion-associated reaction; SAE = serious adverse event

When stratified by infusion rate from 0.9 to < 250.0 mg/hr, IAR, AE, and SAE frequencies decreased significantly with increasing infusion rates (Fig. 4). Similarly, in the < 45 kg subgroup, IAR and AE frequency decreased with increasing infusion rates (both *P* < 0.001), while no significant association between SAE frequency and infusion rate was observed. In the < 30 kg subgroup, the same effect was observed (decreasing IAR and AE frequency with increasing infusion rates; both P < 0.001), and no SAEs were reported in any infusion in this group.

**Fig. 5:**
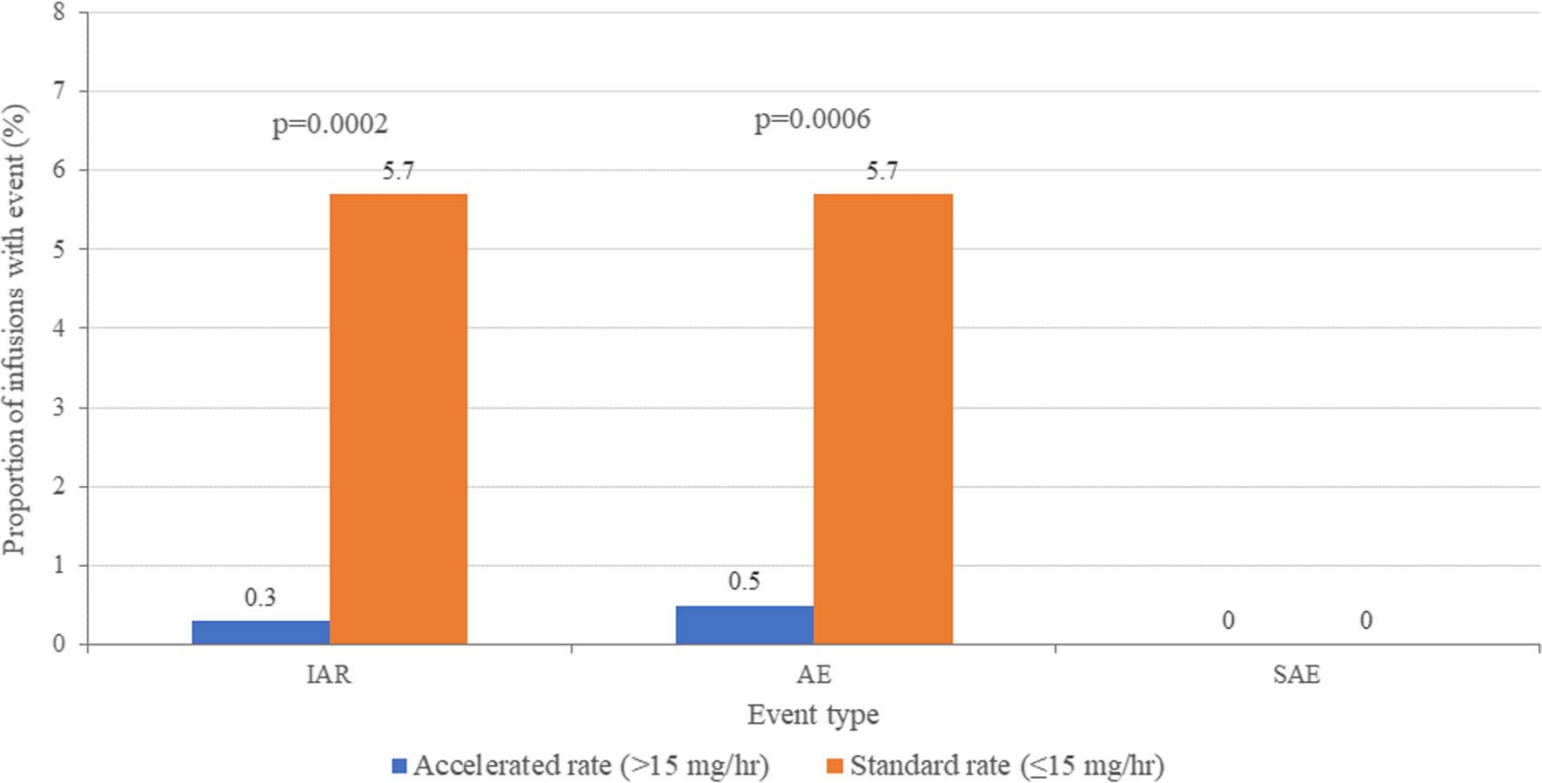
Frequency of IARs, AEs, SAEs in patients < 30 kg according to infusion rate. AE = adverse event; IAR = infusion-associated reaction; SAE = serious adverse event

In the < 30 kg subgroup, AEs and IARs were both significantly less frequent with infusions delivered at an accelerated rate (> 15 mg/hr) compared with those given at the standard ≤ 15 mg/hr rate (*P* = 0.0002 for IARs, *P* = 0.0006 for AEs; Fig. 5). No SAEs were reported in this group.

#### Immunogenicity and results in antibody-positive patients

In patients with anti-agalsidase beta antibody test results available both before and after infusion durations were reduced to < 90 min (n = 9), no changes in antibody status were observed. There were no instances of seroconversion among this group. Consistent with the findings of Tsurumi et al., a significantly greater proportion of patients with positive anti-agalsidase beta antibody tests experienced IARs (41.9% vs 30.7%; *P* = 0.0445) and AEs (61.1% vs 49.3%; P = 0.0497) compared to those with negative tests in the overall population of patients of any weight, although there was no significant difference in the frequency of SAEs (32.3% vs 27.9%; *P* = 0.4546). When the < 45 kg and < 30 kg populations were analyzed separately, no significant differences in safety outcomes according to antibody status were observed.

#### *Infusion characteristics in* < *30 kg subgroup*

Although phenotype and genotype information within the < 30 kg subgroup is not available, the patient characteristics in this group (as seen in Table 1) are indicative of classic Fabry disease (i.e., very early onset of disease manifestation requiring ERT). Five of the 6 patients in the < 30 kg subgroup were male; these male patients all had lower body weights than the female patient, and most were younger (two male patients were 5 years old, and two were 8 years old).

The 6 patients in the < 30 kg subgroup received a total of 326 infusions during the study, 274 (84%) of which were < 90 min. IARs occurred in 6 of the 326 total infusions (< 1%), only two of which were infusions of < 90 min. Four of the 6 patients (all male) received their first infusion in 60 min with no IARs. In the 270 subsequent infusions administered across these 4 patients, only 2 IARs occurred (< 1%), and in 3 of these 4 patients, there were no IARs reported in any subsequent infusion.

## Discussion

Until now, the safety and tolerability of < 90-min agalsidase beta infusions has not been reported beyond a case series published in 2021 by Sanchez et al., in which infusion duration was reduced to 45 min in 6 adult patients (5 females) with Fabry disease after tolerability at a lower infusion rate had been established [22]. In that study, only one IAR occurred across all 6 patients over 20 to 36 months of 45-min infusions, and good tolerability was reported in a male patient with a truncating mutation, a feature commonly linked with classic Fabry disease [22, 25]. Additionally, a prospective, multicenter Italian study has recently been started by Mignani et al. to analyze efficacy, tolerance, and development of anti-drug antibodies with accelerated ERT infusions, the results of which may address several important knowledge gaps in this area [26]. The present post-hoc analysis of a Japanese cohort examined the rates of IARs, AEs, and SAEs in patients receiving infusions of varying durations and infusion rates, including in pediatric patients and antibody-positive patients who are thought to be at greater risk of IARs and for whom a lower rate of infusion is recommended [19, 20, 27].

In the present study, infusions were better tolerated as patients continued receiving ERT; IARs, AEs, and SAEs were uncommon and decreased over the treatment course. In addition, infusions of shorter duration were not associated with increased safety events in any group. Similarly, accelerated infusion rates were not associated with worse safety outcomes in any group; instead, when stratified by infusion rate from 0.9 mg/hr to < 250.0 mg/hr, IAR, AE, and SAE frequencies decreased significantly with increasing infusion rates (Fig. 4). IAR, AE, and SAE rates were all significantly more frequent with infusions delivered at the standard ≤ 15 mg/hr rate vs those given at the accelerated > 15 mg/hr rate in patients < 30 kg.

Despite label recommendations to reduce infusion rates in patients < 30 kg, these low-weight patients were not found to be at higher risk for safety events compared with patients weighing ≥ 30 kg in this study; in fact, no SAEs were reported with any of the 326 total infusions in the < 30 kg group.

Together, these findings suggest that faster infusions are well tolerated not only in the overall Fabry disease population but also in low weight patients of < 45 kg and < 30 kg; thus, the current 15 mg/hr infusion rate cap mandated by both US and EU labels for < 30 kg patients may not be necessary, and infusion rates can be safely increased when done carefully and gradually in patients with established tolerability. In addition, the lack of significant differences in IAR and AE rates between those who received ≥ 90-min and < 90-min infusions suggests that the ≥ 90-min mandate in the US product label may not be necessary once tolerability has been established. It should be noted that reductions of infusion duration should be gradual and performed only in patients who tolerate longer infusions. This is of particular importance in treatment-naïve patients, in whom IARs most frequently occur [28]. In the present study, 76.2% of patients were treatment-naïve, suggesting that, when performed with care, infusion time may be safely reduced even in this higher-risk population.

An important factor to consider when reducing ERT infusion duration is the potential for increased immunogenicity. Due to the observational nature of the present study, only 9 patients had antibody test results available both before and after infusion durations were reduced to < 90 min. Among these 9 patients, no changes in antibody status were observed; however, larger studies designed to prospectively measure the impact of < 90-min infusions on the development of anti-drug antibodies are warranted. Of the 54% of patients who had a positive antibody test at any point during this study, less than half experienced an IAR. While significantly higher rates of IARs and AEs were observed in patients with vs without positive agalsidase beta antibody tests, no significant difference in SAEs was observed. Immunogenicity may be an important contributor to patients’ ERT tolerability; however, the large proportion of antibody-positive patients who were free of IARs and the similarity of SAE rates observed between antibody-positive and antibody-negative patients in this study suggests that there are likely to be other contributing factors.

Most of the limitations of this study were a result of its post-hoc, observational nature. The selection of patients to receive accelerated infusions and the protocol for infusion duration reduction were based on the treating physician’s clinical judgement; thus, a single protocol which may guide other treaters was not used. Studies to prospectively test such protocols are warranted, such as the previously mentioned prospective Italian study that is currently underway by Mignani et al. [26]. Despite this limitation, the results of the present study demonstrate that reducing ERT infusion time using clinical judgement can be safe and well tolerated, even in the absence of a predefined protocol. Assessments of tolerance were performed by the individual treating physicians and were not standardized, and no information on the use of pre-infusion medication was reported, the use of which may affect the incidence of IARs and other AEs. It must be noted that as a post-hoc analysis of an observational study, this study is limited in its ability to assess the impact of reduced-duration infusions on treatment efficacy and long-term outcomes. Future studies that prospectively analyze infusion time reduction protocols and investigate the long-term impacts of reduced-duration infusions on efficacy of ERT, patient compliance, satisfaction, and quality of life are needed. Additional limitations of this study were the single-country design and the small sample size.

## Conclusions

The results of this post-hoc analysis demonstrated no significant difference in safety outcomes with agalsidase beta infusions between patients of different weights. Reduced infusion duration was not associated with worse safety outcomes in any group, nor was accelerated infusion rate. These findings suggest that infusion duration can be safely reduced in patients of all weights if done appropriately. While higher rates of AEs and IARs were observed in antibody-positive patients, there was no difference in the rate of serious events, and the majority of antibody-positive patients still remained free of IARs. Moreover, in patients with available data, no instances of seroconversion to antibody-positive status were observed after infusion durations were reduced to < 90 min.

Given the substantial burden associated with Fabry disease and its treatment, accelerated infusions could be a simple but effective way to confer improvements to patient and caregiver QoL, to reduce healthcare resource use, and to improve patient convenience, which may in turn improve adherence to treatment.

## Data Availability

The datasets used and/or analysed during the current study are available from the corresponding author on reasonable request.

## Abbreviations

AE: Adverse event
ERT: Enzyme replacement therapy
EQ-5D: EuroQol-5 dimension
GL-3: Globotriaosylceramide
IAR: Infusion-associated reaction
SAE: Serious adverse event
SF-36: 36-item short form survey
QoL: Quality of life
